# Professional support during the postpartum period: primiparous mothers’ views on professional services and their expectations, and barriers to utilizing professional help

**DOI:** 10.1186/s12884-020-03087-4

**Published:** 2020-07-11

**Authors:** Yiping Nan, Jingjun Zhang, Anum Nisar, Lanting Huo, Lei Yang, Juan Yin, Duolao Wang, Atif Rahman, Yan Gao, Xiaomei Li

**Affiliations:** 1 School of Nursing, Health Science Centre, Xi’an Jiaotong University, Xi’an, People’s Republic of China; 2 Department of Psychology, Institute of Population and Health Science, Liverpool University, Liverpool, UK; 3 Department of Biostatistics Tropical Clinical Trails Unit, Liverpool School of Tropical Medicine, Liverpool, UK; 4 Department of the Second Affiliated Hospital of Xi’an Medical University, Xi’an, China

**Keywords:** Primiparous mothers, Professional support, Maternal and child services, Utilizing support, Needs

## Abstract

**Background:**

Primiparous mothers who lack of experience and knowledge of child caring, are usually overwhelmed by multifarious stressors and challenges. Although professional support is needed for primiparas, there is a gap between the necessary high-quality services and the currently provided poor services. This study aimed to explore Chinese primiparous mothers’ views on professional services, identify barriers to utilizing professional support, and further understand mothers’ expectations of and preferences for the delivery of professional services.

**Method:**

A descriptive phenomenological study design was utilized in this study, and semi-structured interviews were conducted with 28 primiparous mothers who had given birth in the first year period before the interview and were selected from two community health centres in Xi’an city, Shaanxi Province, Northwest China. Each conversational interview lasted between 20 and 86 min. Colaizzi’s seven-step phenomenological approach was used to analyse the data.

**Results:**

Three major themes were identified: (a) dissatisfaction with current professional services for postpartum mothers, (b) likelihood of health care professional help-seeking behaviour, (c) highlighting the demands for new health care services. The related seven sub-themes included being disappointed with current hospital services; distrusting services provided by community health centres, private institutes and commercial online platforms; preferring not seeking help from professionals as their first choice; hesitating to express their inner discourse to professionals; following confinement requirement and family burden prevents mothers from seeking professional help; experiencing urgent needs for new baby-care-related services; and determining the importance of mothers’ needs. The necessity of professional support in the first month after childbirth was strongly emphasized by the participants. Online professional guidance and support were perceived as the best way to receive services in this study.

**Conclusion:**

The results of this descriptive phenomenological study suggested that the current maternal and child health care services were insufficient and could not meet primiparous mothers’ need. The results also indicated that identifying barriers and providing services focused on mothers’ needs may be an effective strategy to enhance primiparous mothers’ well-being, and further suggested that feasibility, convenience, and the cultural adaptability of health care services should be considered during the delivery of postpartum interventions.

## Background

Childbirth and the postpartum experience often generate many physiological and emotional changes for mothers, new motherhood can be an overwhelming experience due to mothers experiencing a multitude of stressors, including routine baby care, physically taxing household duties, a lack of sleep, and breastfeeding difficulties [1–3]. This situation makes the postpartum period a very challenging time for mothers, especially for primiparous mothers who lack previous knowledge and experience [4]. These challenges can even result in mental health issues, including post-traumatic stress disorder, anxiety, and depression [2, 3, 5]. Postpartum depression (PPD) is the most common psychiatric illness following childbirth, is mostly likely to occur anytime during the first year after delivery and poses a major global public health challenge [6–8].

Furthermore, it can be difficult for primiparous mothers to adjust to their new role and to shoulder the responsibility of caring for their babies [9, 10]. Primiparous mothers often feel frustrated and unsure due to, for example, being unable to identify any specific reason for their babies’ frequent crying [11]. Therefore, most postpartum new mothers expressed their eager demands for professional guidance and support with their baby care and their own care [12].

Strong evidence suggests that social support benefits new mothers greatly [13, 14]. For example, empirical studies have consistently shown that adequate support can reduce stress and the risk of developing depression and can increase parental sensitivity and feelings of self-worth [15, 16]. One of the main sources of social support is professional support, which can be provided by many professionals in different areas, including midwives, doctors, and nurses among others [4]. The professional services provided take different forms and are efficient, for example, postnatal care interventions such as home visits by midwives or public health nurses, phone-based support programmes, and online consultation have been shown to help improve maternal confidence, increase successful breastfeeding, and decrease postpartum fatigue and depression [17–20]. Mothers particularly value professionals who focus on their needs and the development of trusting relationships, including the opportunity to develop new skills in problem solving [21–23].

Although support from professionals is urgently needed for new mothers and has been proven to be quite effective, there is a gap between the necessary high-quality services and the poor services that are currently provided. Many countries have similar problems, when discussing their current views on postnatal care, new mothers expressed great disappointment with their experience of the quality of postnatal care, including health care professionals spending insufficient time answering their questions, providing information with little patience and few explanations, and delivering insufficient care [24, 25]. Unsatisfied mothers also emphasized that hospital staffs were unconcerned and unsympathetic towards their and their infants’ needs. They reported that caregivers were unfriendly, disrespectful, and impersonal [26]. In addition, early discharge from the hospital made dissatisfied mothers feel insecure and worried, and the absence of home visits also magnified these feelings [13]. Negative comments were also related to the fact that the services provided did not correspond to the care requirements [27, 28]. Professionals usually only focused on the physical aspects of baby care but neglected the emotional needs of mothers [29, 30]. This situation may be due to a shortage of health care professionals and to restrained resources [31, 32]. However, one of the greatest barriers to providing appropriate care and support is the lack of identification of mothers’ needs [33]. This situation is particularly prominent in China [34]. Therefore, it is crucial to explore Chinese new mothers’ views and expectations of professional support.

Although several qualitative studies on postpartum mothers’ needs have been conducted in Western countries, the evidence obtained from those studies may not be applicable for Chinese mothers [35]. Numerous studies have identified the cultural differences in risk factors for and manifestations of PPD symptoms between the Western and Asian countries [36–38]. For example, Chan & Williamson noted that in contrast with their Australian counterparts who attributed their depression to feelings of incompetence as an ideal mother and guilt, Chinese mothers expressed much anger with their husbands and mothers-in-law and attributed their PPD to the low quality of those relationships [39]. Several distinct cultural factors connected with childbirth have been identified in China, such as, discrimination with respect to the sex of babies (preference for a son), the practice of “sitting the month” (“zuo yue zi”, a common Chinese practice of confinement during the first month postpartum and the strict observance of certain specific dietary rules and behavioral taboos), and the custom in which the mother-in-law cares for the newborns baby [35, 40, 41]. As a result, given the impacts on mothers’ perspectives towards professional support, it is necessary to concern cultural factors when providing support to postpartum mothers.

There is a lack of available literature on the views of postpartum mothers on professional support, barriers and needs in China. In addition, most previous local studies of depressed or at-risk mothers ignored the experience of healthy mothers.

Therefore, considering the aforementioned literature gaps, this study focused on the postpartum period and aimed to explore primiparous mothers’ views on professional services, identify barriers to mobilizing professional support, and further understand their expectations of and preferences for the delivery of professional services.

## Methods

### Design

A descriptive phenomenological approach was utilized in this study to explore primiparous mothers’ views on professional services, their expectations, and perceived barriers to obtaining professional help. This approach was chosen to provide an accurate description of the phenomena examined [42]. Descriptive phenomenology originated from Husserl’s (1960) work and is commonly used in nursing and midwifery research [43]. Husserl contended that the object of scientific study is the phenomenon perceived by the individual’s consciousness. Thus, no assumptions, philosophical or scientific theories, logical procedures, other empirical science or psychological speculations should inform phenomenological inquiry [44]. In Husserl’s descriptive approach, researchers are required to focus on participants’ experience of a phenomenon and identify the essences of the phenomenon while suspending their own beliefs, attitudes, previous experience and assumptions [45]. Thus, with the guidance of the methodology above, the researchers aimed to maintain openness, question their preconceptions and adopt reflective attitudes during the entire study process, including data collection, data analysis and reporting.

### Study sites and participants

The study was conducted at two community health centres in Xi’an City, Shaanxi Province. The directors of those two health care centres assisted the researchers to deliver the invitation letters to potential participants who were recruited for the study when they went to community health care centres for postnatal care. The sample size was determined based on the data saturation principle. A purposive sampling method was utilized with the inclusive criteria as 18 years old or above, had given birth to their first baby, were within the first year postpartum, and expressed interest in participating in the study. A strategy to maximize the variation in the sample was employed when selecting the participants. Women from different age groups, education levels, economic status and postpartum periods were sought to increase variation. A total of 40 potential participants were selected, while data collection was terminated after the 28th participant as data saturation had been achieved.

### Ethical considerations

Ethical approval was obtained from the Ethics Board of the Health Centre of Xi’an Jiaotong University. The participants were well-informed the purpose, methodology, procedures, benefits and potential risks of the study, and the written informed consent were obtained from all the participants. They were also informed that they could withdraw anytime during the interview and the audio recordings and transcripts would be stored in encrypted databases at the School of Nursing to guarantee the confidentiality of the information.

### Data collection

The two researchers (YP, N & JJ, Z) conducted private individual face-to-face interviews with all the participants in the health education rooms of the two community health care centres. During the entire process, the first researcher performed the interviews, and the second researcher recorded the interviews, took notes, made summaries, and supplemented the data as necessary.

The interview questions were obtained and confirmed through a review of the literature and discussions with the study team members and were further modified after consulting with a gynaecologist, a sociologist and a psychologist who were experienced in maternal health care, qualitative research method and the maternal mental health. A pilot interview of three participants was conducted for further revision of the questions to guarantee their suitability and acceptability. Finally, eight open-ended questions were developed to guide the interview. The questions are outlined in Table 1.

**Table 1:** Guidance for the participants interviews

Additional questions were asked flexibly depending on the responses of the participants. The prompt questions for each theme were developed to be nonprescriptive and open-ended.	
1. What do you think about the current medical institutions, such as hospitals, community health centres, private clinics or other institutions in your resident areas?	
2. Could you please tell us about your experience of seeking help from health care professionals in the above-mentioned health care institutions?	
3. Do you like to seek help from health care professionals when you are in trouble? Why?	
4. Are there any difficulties or barriers when you need health care professional support? Please give us examples.	
5. Based on your experience, in what kind of situations or difficulties would you require assistance from health care professionals? In what kind of situations or difficulties would you not need any professionals’ help? Why? Please provide some examples.	
6. Please describe which health care professionals you need most after childbirth and please specify the services that you need most. Why?	
7. Do you have any recommendations for current health care professionals? What are your expectations for new health care services? Why?	
8. Please identify which method of health care service delivery that you prefer from health care professionals?	

After the interview, the participants were required to complete a demographic questionnaire. All of the transcripts were sent back to the participants, and follow-up phone calls were made with a few participants whose views were ambiguous to check the accuracy of the information.

### Data analysis

Colaizzi’s seven-step phenomenological approach was applied to analyse the data [46]. The interviews were transcribed verbatim in Chinese within 24 h after the interviews by the two bilingual researchers who collected the data. All of the transcripts were then cross-checked against the audio records by the two researchers above. The entire data analysis process was conducted in Chinese. The two researchers (YP, N & JJ, Z) used the seven steps approach for this study as follows: 1. They read each transcript carefully, and combined with the field-notes taken in the interviews to obtain a more accurate understanding of the descriptions. 2. They extracted meaningful statements related to the participants’ views and the barriers of and expectations for accessing professional services. 3. They coded significant statements and labelled the statements with the participants’ keywords and phrases, and cross-checked and discussed all the codes. 4. They repeated and checked the first three steps, and each code was read, evaluated repeatedly and deliberately, and then clustered together into multidimensional categories. 5. Seven sub-themes were identified, and exhaustive descriptions were developed. 6. Each final theme was determined based on continuous discussion, comparison, reintegration, and inspection of the sub-themes among all team members. 7. All of the transcripts were returned to the participants to check their accuracy in capturing the participants’ intended meaning.

To maintain the openness and confirmability of the data analysis, the interviewees’ accounts were given priority to help the authors understand their experiences and stories in the participants’ own words. The sub-themes and themes were generated from the data and were not based on a preidentified framework. In addition, all discrepancies that arose during the data analysis process were discussed and clarified by the two researchers mentioned above and a third researcher until consensus was achieved.

The emerging themes were discussed as a team to determine whether to retain them and to ensure the consistency and balance of the main theme.

## Results

In total 28 primiparous mothers who were aged between 24 to 40 years (30.08 ±3.43) completed the study. Seventeen mothers had a bachelor’s degree or higher education level. The average age of the babies was approximately 5 months (5.56 ±3.23). Further characteristics of the participants are presented in Table 2. The duration of the interviews ranged from 20 to 86 min (mean = 32 min). Data analysis resulted in the identification of three themes and seven sub-themes, which are shown in Table 3.

**Table 2:** Characteristics of the Mothers Interviewed (*n* = 28)

Characteristics	n (%)/ $\overline{x}\pm \mathrm{S}$
**Age**	
(year)	30.08 ±3.43
**BMI**	
< 25 kg/m^2^	23 (82.1%)
≥ 25 kg/m^2^	5 (17.9%)
**Residence**	
Urban	24 (85.7%)
Rural	4 (14.3%)
**Education level**	
High school or below	4 (14.3%)
Junior college	7 (25.0%)
Bachelor degree or above	17 (60.7%)
**Employment status**	
Full-time job	14 (50.0%)
Unemployed	14 (50.0%)
**The way of delivery**	
Vaginal delivery	12 (42.9%)
Cesarean delivery	16 (57.1%)
**Age of baby at interview**	
(month)	5.56 ±3.23
**Gender of their baby**	
Boy	15 (53.6%)
Girl	13 (46.4%)
**Infant illness within 4 weeks after childbirth**	
Yes	2 (7.1%)
No	26 (92.9%)
**History of depression**	
Yes	8 (28.6%)
No	20 (71.4%)
**Family per capita income**	
< 4000 yuan	12 (43.9%)
≥ 4000 yuan	16 (57.1%)

**Table 3:** Themes and Sub-themes Identified in the Interviews

**Themes and sub-themes**	
1. **Dissatisfaction with current professional services for postpartum mothers**	
Being disappointed with current hospital maternal and child health care services.	
Distrusting services provided by community health centres, private institutes and commercial online platforms.	
2. **Likelihood of health care professional help- seeking behaviour**	
Preferring to not seek help from health care professionals as their first choice.	
Hesitating to express their inner discourse to health care professionals.	
Following confinement requirement and family burden prevents mothers from seeking professional help.	
3. **Highlighting demands for new health care services**	
Experiencing urgent needs for new baby-care-related health care services.	
Determining the importance of mothers’ needs.	

### Dissatisfaction with current professional services for postpartum mothers

The vast majority of mothers in this study indicated a low level of acceptance and satisfaction with local maternal and child health care. Their comments regarding health care institutions for maternal and child health care can be categorized into two themes: (a) being disappointed with current hospital maternal and child health care services and (b) distrusting services provided by community health centres, private institutes and commercial online platforms.

### Being disappointed with current hospital maternal and child health care services

Many mothers mentioned that the services provided in hospital maternity settings during the postpartum period were quite insufficient; they also claimed that health care professionals usually only focused on their current physical health issues, such as prolonged lochia and others. Health care professionals rarely considered the mothers’ long-term psychological, emotional and informational needs after discharge. Mother 28 emphasized her dissatisfaction with the hospital services.

*“The service provided in hospitals is totally insufficient. I think the postpartum period is very important … We should have received some related education about postpartum problems before we gave birth, but we didn’t … Most of the time, the provided service was just superficial; they don’t take it seriously … what they did for us is not enough.” (Participant 28)*

Furthermore, participants complained that the postpartum hospitalization time was very short and that follow-up checks after discharge were either absent or very simple. Some mothers noted that even though there was a follow-up, health care professionals often made a simple phone call without a home visit. Many mothers felt neglected and uncared for by health care professionals because they felt that they did not receive enough formal support from the hospitals. Mothers 5 and 27 both stressed the importance of follow-up visits by health care professionals. As mother 27 recounted,

*“I had not received any service after discharge from the hospital, but I believe that follow-up visits are very important. For example, my brother’s wife had an episiotomy. Afterwards, the stiches were not taken out completely. But who knew that? The wound was infected with pus eventually. Then, she went to see the doctor by herself. There was no one who reminded her of such things that may occur after surgery.” (Participant 27)*

Several mothers mentioned that the paediatric departments in their local government tertiary hospitals were very busy, and the doctors and nurses there were time-constrained and often impatient. Some mothers indicated that the health care professionals in the government tertiary hospitals did not even fully listen to their complaints and did not want to talk to them as those health care professionals did not have enough time. The mothers felt that they did not receive adequate respect. One mother felt upset when she took her baby to go to visit the paediatrician:

*“After all, there are so many patients in big hospitals. When I have a little problem about my child, I really need to consult a family doctor or GP … but there was none, so I had to wait in a long line in the big hospital for a long, long time…when I eventually saw the doctor, the length of the conversation was no more than five minutes … I wish that health care professionals could be more patient and spend more time on every baby’s examination.” (Participant 13)*

Some mothers also reported that they were treated with a lack of compassion and in these large hospitals, efficiency and profit were regarded as priorities over patients’ needs.

*“My daughter has a red spot on her face, I am very worried … I couldn’t figure out what happened. During her examination, my baby cried fiercely … I was very nervous, but the doctor didn’t care about our emotions at all, he gave no comfort to us, and didn’t explain my daughter’s condition in detail either.” (Participant 23)*

### Distrusting services provided by community health centres, private institutes and commercial online platforms

Most of the participants indicated that they nevertheless trust the government tertiary hospitals rather than other institutions, because of the high professional skills levels and well-equipped facilities in those hospitals. Some mothers expressed their hesitation to seek help from health care professionals in community health centres because they felt that doctors and nurses in these settings had lower qualifications, professional titles and skill levels than in tertiary hospitals. Furthermore, some mothers also mentioned that the poorly equipped community health care centres could not guarantee the reliability of the examination results. Mother 2 expressed her feelings about the community health centres:

*“I think the community health centres are not so good … their function is not specific enough. Only when my child needs to be vaccinated, would I go there … I am not confident in such settings because I think health workers there can’t be as professional as in the big hospitals… and the examination equipment there is less reassuring … I feel that community health centres should play a role in consulting and being the primary treatment provider. But in fact, I get little help from there.” (Participant 2)*

Two mothers mentioned that private institutes did not have enough qualified health care professionals and were profit-driven*.* For example, some private clinics have lactation masseuses who help mother with lactation by using Chinese massage. The mothers indicated that they were sceptical about those professionals’ educational backgrounds and professional certificates. In addition, the costs of the services were often too expensive for mothers to afford. Mother 28 recalled her experience finding help for her difficulty breastfeeding:

*“Low milk supply is a big problem for me; it troubled me a lot. I really needed a professional who could help me at that time. But it’s difficult to find a qualified lactation masseuse in a formal way; those in the private clinics are inexperienced and not well-trained most of time” (Participant 28)*

Many participants had mentioned that online services brought a great convenience to their life and had advantages over traditional ways. However, some of the participants were doubtful of the accuracy and reliability of information provided on many profit-driven online platforms, and some mothers reported that ample educational information was tied to the sales of goods, which may confuse or mislead consumers. Furthermore, some mothers expressed their dislikes towards advertisements and promotions on such platforms. Moreover, these mothers also expressed concerns with the qualifications of the purported professionals who provided online services. Therefore, many participants expressed expectations for new non-profit online platforms which were officially operated by the government or government tertiary hospitals to which they could easily access.

*“I often consulted doctors online on baby health issues. To be honest, I didn’t trust them (the doctors) so much … but I had no choice, so I often combined their advice with information from other sources.” (Participant 21)*

### Likelihood of health care professional help-seeking behaviour

Some mothers expressed that their willingness to seek professional help is not very strong unless faced with acute or critical conditions of their own or their babies. The participants’ professional help seeking behaviour was summarized into three sub-themes: (a) preferring not to seek help from health care professionals as their first choice, (b) hesitating to express their inner discourse to health care professionals, and (c) following confinement requirement and family burden prevents mothers from seeking professional help.

### Preferring not to seek help from health care professionals as their first choice

Several participants indicated that they were used to solve common problems by themselves instead of mobilizing support from health care professionals. Some mothers stressed independent values and thought that it was a sign of independence to deal with most of the problems by themselves. One mother mentioned that she felt embarrassed to discuss her private issues, especially emotional and mental issues with any other people, including health care professionals, because she neither wanted to be a burden to others and nor wanted to show any signs of vulnerability.

*“I thought … It was irresponsible to expose my negative feelings to others … which may have a negative influence on their lives. It's just like throwing my own garbage to someone else … neither I nor anyone else will feel well from that.” (Participant 23)*

In addition, most of the participants emphasized that their problems that occurred frequently in daily life were generally minor ones, so they usually gave priority to the most convenient and economic ways to solve such problems. Most of the participants mentioned that they had benefited from using professional online platforms, which were perceived as the most convenient way to obtain information. The standard forms of such services included apps, videos, online forums, and online counselling. Professional none-profit apps were the most appreciated form by the majority of participants, who preferred, trusted, and spoke highly of them because of their convenience, many functions, and lack of time and space constrains, which allowed mothers to access information anywhere or anytime. Most of the time, participants believed that almost all of their problems could be solved in that way.

*“For our office staff, face-to-face services are out of date; the internet platform is faster… I could take advantage of the short time of a bathroom break or other break to read messages and communicate with somebody through apps.” (Participant 13)*

Compared to ask for health care professional help, many mothers in this study were more likely to discuss current issues with reliable people around them, such as their own parents, significant others or peers. The mother’s parents, especially their mothers, were mentioned as a trustworthy source of emotional support; the participants noted that their mothers made great efforts for their daughters’ families and often expressed understanding and sympathy of their daughters’ suffering and distress. Some of the participants believed that sharing with peers was also effective and that peers could be trusted; sharing with peers was described as powerful and as imparting cognitive empathy for the roles of different mothers. Mother 5 showed her appreciation for support from peers:

*“I benefited from peer groups a lot because everyone can obtain limited information, but by exchanging messages and sharing experiences, and I could view one issue from various perspectives … If I was lucky enough, I could find the information that just matched my requirements.” (Participant 5)*

### Hesitating to express their inner discourse to health care professionals

With the influence of “saving face” culture in China (saving face, refers to the social confidence in individuals’ moral character in society and that people cannot properly function in society if the integrity of this character is broken [47]), mothers stated that seeking help from mental health professionals for inconsequential matters in daily life was unnecessary. The mothers worried that if they sought professionals help for their family conflicts, the prejudices or stigma of making family life public would harm themselves and their family members. One participant even preferred discussing matters with a stranger rather than a health care professional:

*“When I was upset, I hadn’t considered turning to health care professionals yet … I think it would make more sense and be safer to discuss my story with a stranger.” (Participant 23)*

Family conflicts were sensitive topics for almost all the mothers in the study, and the conflicts between them and their mothers-in-law were mentioned as the main reason for their distress. However, most of the mothers believed in the old Chinese adage that domestic shame should not be made public no matter what happens, and it should be solved within the family. If others knew about the conflicts in their families, it would have made the mothers feel ashamed and would have also demonstrated their incompetence at becoming good mothers. Thus, most of the time when the mothers experienced difficulties, turning to health care professionals was not their first choice. As Mother 4 said,

*“Actually, I just let bad emotions blow over, and I would feel better… after all, the fact is that we are family … and no matter how many conflicts happen, they can be settled down eventually … It is not necessary to seriously take them into consideration.” (Participant 4)*

Additionally, even some mothers suffered substantially from family conflicts, they supposed that such conflicts were universal phenomena and therefore followed social norms of keeping family conflicts inside without any effort to solve it. Mother 23 related her point of view:

*“There is an old saying in China, “even an upright official finds it hard to settle family quarrels”. I think that even my mother-in-law has many problems with me, but we are one family… I believe conflicts happen in every family… I think I could endure that.” (Participant 23)*

Some mothers decided to hide their own feelings from others because of their previous unsuccessful experiences of help-seeking. These mothers felt disappointed because their closest family members could not fully understand them. They also felt frustrated with the ineffective communication by the people around them, including health care professionals. The participants tended to believe that without the same experience, it is impossible for others to understand their feelings, or show sympathy for them, let alone professionals who were not familiar with them at all.

*“Unlike other young mothers, I was very old when I gave birth … I suffered a lot from the birth and felt very uncomfortable after that … But when I shared my feelings with peers, they just told me it was a kind of neurosis … the doctors didn’t treat my complaints seriously either and thought my body was alright … they couldn’t understand me at all. It was useless to discuss my feelings with them.” (Participant 5)*

### Following confinement requirement and family burden prevent mothers from seeking professional help

Because of the mothers’ weakened physical condition during the first month of confinement “yue zi” (which is a postpartum tradition among Chinese mothers in which new mothers are required to strictly comply with specific regulations for 30 or 40 days postpartum [11]), it was inconvenient for mothers to leave the house. As Mother 27 said:

*“He (the baby) is too small to go to crowded places, especially hospitals, due to fears that he will get sick… It was also inconvenient for me to go outside with him. I was still in confinement at that time (in “yue zi”). I was not allowed to go outside” (Participant 27)*

In addition, many mothers expressed that they often tried to mobilize support from health care professionals, but several mothers showed their hesitation towards the cost of money and time on seeking professional support. In addition, some of the mothers expressed that they had been overburdened with baby care and housework after confinement, which caused that they were too tired to go outside most of the time. These mothers also felt there was no time at all to consider their own feelings. If they experienced negative emotions, they would put them aside to do housework or to play with their babies. Mother 6 recounted,

*“My life was filled with various household chores… I was too tired to think too much about my distress… I hung out with my baby in the morning, had a nap at noon, and played with my baby in the afternoon. When the day finished, I had forgotten my distress, especially when faced with my baby’s smile.” (Participant 6)*

### Highlighting demands for new health care services

Twenty-one mothers emphasized that professional support was urgently needed and that they had high expectations for new health care services. Their demands were classified into two aspects: (a) experiencing urgent needs for new baby-care-related health care services and (b) determining the importance of mothers’ needs.

### Experiencing urgent needs for new baby-care-related health care services

Baby-care-related professionals, such as lactation masseuses, nutritionists, paediatricians, and nurses, were mentioned repeatedly by many mothers, in order to help them with breastfeeding, complementary food supplements, preliminary diagnosis of their babies’ diseases, and training of skills for baby care.

*“When I added complementary food for my boy, I really didn’t know which kind of food was suitable for him and whether the nutrition was right or reasonable. I always worried, not only about if he had indigestion but also about malnutrition. I really needed some professional advice.” (Participant 26)*

Considering their reliance on large tertiary hospitals, several mothers indicated their desire for hospital hotlines that would provide round-the-clock counselling for when their babies got sick. In addition, some mothers wanted to have regular appointments with experienced doctors or nurses about their babies’ existing problems at a convenient place near their home. Mother 15 had the following expectations for health care professionals:

*“In fact, it is too troublesome to take children to the hospital all the time. The community health centre is very convenient but not reliable, the most important parts are the health care professionals who provide services. Rich clinical experience and knowledge and proficient caring skills are the key points for professionals.” (Participant 15)*

The majority of mothers also mentioned their requirements for baby-care-related knowledge and information because they believed that acquiring more knowledge in advance would help them have a better understanding of their babies’ needs and would be good for the health of their babies. This knowledge would also allow greater anticipation of possible risks and the establishment of adequate preparations for the risks. The requirements for knowledge could be divided into several aspects as follows: instructions regarding the growth, development and care of babies; methods to promote babies’ growth and development; and possible events that might occur during babies’ growth.

*“I really confused on her (my daughter’s) mental development. I have little knowledge on the psychological development of children, but I know there must be some meaning in her emotions and actions. I just try to understand her by relying on my intuition. I need professional guidance.” (Participant 22)*

The early identification of symptoms of diseases and the management of minor problems were also strongly emphasized by many mothers. Finally, four mothers emphasized the importance of targeted guidance through the provision of information and knowledge according to their baby’s different stages of the growth. These mothers wished that they could receive reminders and cautionary messages for several key periods during their babies’ growth. As Mother 20 said,

*“I wish there were some tips from health care professionals to regularly remind me of important things, for example, vaccinations and examinations. What’s more, I would like to receive basic information about growth and development standards that is matched with my baby’s growth stage. For example, at what age can the baby crawl or sit?” (Participant 20)*

### Determining the importance of mothers’ needs

Several mothers reported their personal needs for physical and mental recovery during the postpartum period, including guidance for achieving physical fitness, having a healthy diet, and regulating their emotions. Therefore, related professionals, such as weight management doctors, nutritionists, and psychologists, were mentioned by the mothers as necessary to meet their postpartum requirements. However, some mothers stressed the shortage of the professionals in those areas. One mother demonstrated her keen expectations for professional help:

*“I just want to say that it doesn’t matter if professionals couldn’t offer perfect services; it is also acceptable if there is someone who just provides the information about how to get access to the professionals above. Most of the time when I needed help, my mind went blank; I didn’t know who I should turn to, who could be trusted.” (Participant 28)*

The provision of professional support during “yue zi” was emphasized by many mothers. Due to this widely accepted social custom, mothers were restricted from leaving the house after giving birth. Therefore, mothers expressed their urgent needs for obstetricians and nurses to help them identify abnormal postpartum symptoms, specifically related to lochia and wounds, and to teach them interventions to prevent infection and reduce wound pain.

*“I had a vaginal incision while giving birth; there were doctors and nurses who checked and nursed my incision and lochia on time when I was in the hospital, but when I left to come home, there was nobody who could help me with that anymore. For example, I was not sure whether the incision was infected or not when I felt unwell, but I couldn’t check it by myself.” (Participant 28)*

In addition, some mothers stressed the necessity of receiving health education. These mothers hoped to have access to reliable scientific knowledge to help them achieve a better recovery. The delivery of instructions via various methods was also needed to remind them of appointments after delivery.

*“I was in a confinement (yue zi) service centre for a month; I indeed received much service in my own recovery, but when I left there, there was no one to guide me anymore. I didn’t know how to go on recovering and needed relevant knowledge at least.” (Participant 10)*

Although psychological consultants were mentioned by some individuals, it seemed that most mothers were sensitive regarding that topic. Some participants explained that they just needed to find somebody to talk to or to receive mental health guidance from professionals rather than psychologists. Mother 13 felt it was not necessary to see a psychologist:

*“I think chatting with health professionals like nurse is enough; I feel much better after I talked with you in so much detail. In fact, those things were unworthy to mention to psychologists; that’s too exaggerated.” (Participant 13)*

Finally, support from professionals to improve family relationships was mentioned by three mothers.

## Discussion

This descriptive phenomenological study aimed to explore primiparous mothers’ views on professional services, their perceived barriers to utilizing professional support, and their expectations of professional support. The findings of this study showed primiparous mothers’ negative attitudes towards current maternal and child services and professionals. Disappointment and dissatisfaction of primiparous mothers were revealed in many areas, including early discharge from the hospital, simple or absent subsequent follow-up, and little consideration for long-term and potential problems. The participants also believed they were treated with uncaring manners by time-constrained hospital health care professionals who were not patient enough and seemed unconcerned about mothers’ feelings and needs. These results were highly consistent with findings in other countries [11, 26]. It may be explained by the fact that there is not enough qualified health care professionals and enough beds in large government hospitals [48]. These results imply that increasing investment on the numbers of health care professionals and medical resources is the preconditions for improving the quality of services and service satisfaction of mothers [49].

Participants manifested that other health care institutions such as community health centres and private institutes, were not trustworthy because of their poor equipment, less experienced and less qualified health care professionals, and high costs. This finding suggests that standardized professional training and financial input should be offered to primary medical facilities to improve their service quality and further complement hospital health care services [50–52].

The likelihood of mothers’ professional help-seeking behaviour was partially influenced by the circumstances of the current Chinese health care system. Many participants in this study expressed that they seek help from their parents, peers, online platforms or handle the problems by themselves, rather than seeking professional help. This may be partially due to the facts that they didn’t know what the results or impact of the health problems they faced [53]. The result also indicated that mothers’ unwillingness to seek professional help on mental health problems related to family conflicts might be due to stigma towards them, similar results could be found in many previous studies [54–56]. Therefore, future interventions were needed to improve knowledge levels and reduce stigma of mothers in order to change their attitudes and behaviour regarding professional services [57–59].

Additionally, cultural differences largely influence the preferred types of support. Unlike individualistic Western culture, Asian culture is more collectivistic, and indirect support from other people giving companionship and attentiveness is preferred [60]. Therefore, cultural differences may account for the mothers’ help seeking preference and behaviour in this study. This finding illustrated that the Chinses cultural context should be considered when providing support to mothers, furthermore, peer supports could be effective for solving mothers’ problems.

Regarding other acceptable type of support, non-profit online platforms were considered as the most popular and convenient support method by the majority of mothers. Due to the development of technology and network processes, the use of technology in postnatal care has been proven to be efficient and satisfactory in many studies [61]. The participants in this study perceived non-profit apps to be good means of receiving help and support because of their multi-functionality and convenience. Previous studies have found successful results with app-based interventions [25, 62]. However, the shortcomings of current apps and other online approaches were evident, including complicated contents, unclear classified navigation, too much irrelevant information, and information with little scientific background, as reported in other studies [61]. Thus, it is important to overcome the disadvantages listed above and to develop more professional, well-organized, integrative, useful and clear apps or other software [63].

Some mothers hesitated to speak out their family conflicts and showed less willingness to seek-help from professionals for emotional problems. The “saving face” Chinses culture may explain this finding. “Saving face” is a typical cultural phenomenon observed throughout China [64]. Due to its profound influence, Chinese people may consider family conflicts and related mental health problems to be shameful and assume that they would lead to gossip and convey a negative impression of their entire family to others. This cultural context was also proved in other studies in which Asian people held family and social relationships in higher value and in which mothers were regarded as the weaker sex and had a greater tendency to rely on others, especially on their family members and partners [61]. The results indicated that cultural factors, such as saving face, should also be considered when conducting interventions in China.

The first month of confinement and its effects was emphasized by many mothers in this study. Home visits, health assessment and care were usually absent, with very few channels or difficult access to professional help. These phenomena had also been observed in other countries, such as America, where mothers receive little professional guidance in the 6 weeks after delivering a baby [65]. Therefore, multiple professional services should be provided during the first months postpartum [11, 66].

Mothers clearly expressed their wishes for new health professional services. Most of their needs were the same as mothers’ needs mentioned in other studies, including urgent needs for knowledge delivery, skills training, more access to different kinds of professionals, and more comprehensive service systems [26]. It is worth noting that the timing of service delivery was highly emphasized in this study. Participants hoped to receive enough guidance before giving birth, and they wished that services could be provided at several critical times corresponding to their and their infants’ demands. To counteract the current confusion caused by large amounts of chaotic information, they also wanted to be provided with tips to remind them of important points for their own and their babies’ health.

Some participants indicated that face-to-face professional services were irreplaceable for providing medical services and many mothers expressed their needs for home visits and consultations given by experienced and qualified doctors or nurses during the first month of confinement. However, with the current health care workforce and expense, it is difficult to meet all the above needs [67]. Therefore, Considering the preference for online services and mother’s health visits needs mentioned in this study, the most practical ways to provide postpartum services may be a combination of the online and face-to-face methods, which needs to be further verified in future studies. As Nicole E. Pugh successfully found that a therapist-assisted internet-delivered intervention was effective and had greater advantages in reducing attrition and improving adherence [68].

### Limitations

This study has a few limitations. First, most participants came from cities, with few participants from the countryside; consequently, the results of this study may be subject to selection bias. Further exploration with mothers of different ethnicities and from different districts would be beneficial to expand the findings of this study. Second, participants within the first postpartum year were selected and were asked to share their experiences retrospectively, which may have led to recalling bias. It should be noticed that the data were from subjective perspectives of the participants, indicating that future studies are needed by mix-method to fully understand primiparous mothers’ experience from both the subjective and objective perspectives.

## Conclusion

Despite the limitations of the study design, this research provided insights into primiparous mothers’ views on current health care professional support, expectations for new services and the barriers to mobilizing health care professional support. The results of this study implied that the current maternal and child health care services were insufficient and could not meet the needs of primiparous mothers. The barriers, and preference identified in this study could help the health care professionals with a better understanding of the primiparous mothers’ professional help-seeking behaviour. The results also indicated that providing services focused on mothers’ needs may be an effective strategy to enhance primiparous mothers’ well-being, and also suggested that feasibility, convenience, and the cultural adaptability of health care services should be considered during the delivery of postpartum interventions.

## Data Availability

All data used by or generated in this study are available from the corresponding author upon reasonable request.

## Abbreviations

PPD: Postpartum Depression
GP: General Practitioner
